# Successful conservative management of a colorenal fistula complicating percutaneous cryoablation of renal tumors: a case report

**DOI:** 10.1186/1752-1947-6-365

**Published:** 2012-10-26

**Authors:** Amir IS Morgan, Andrew Doble, R Justin Davies

**Affiliations:** 1 Cambridge Colorectal Unit and Department of Urology, Addenbrooke’s Hospital, Cambridge University Hospitals NHS Foundation Trust, Hills Road, Cambridge, CB2 0QQ, UK

**Keywords:** Colorenal fistula, Cryoablation, Renal cell carcinoma

## Abstract

**Introduction:**

Colorenal fistula is a rare phenomenon and may complicate percutaneous cryoablation of renal cell carcinoma. Treatment remains controversial.

**Case presentation:**

A 62-year-old Caucasian man presented with pneumaturia and left flank pain six weeks following ultrasound-guided percutaneous cryoablation of two recurrent lesions in the left kidney 14 years after partial left nephrectomy for a left renal cell carcinoma. A computed tomography scan eight weeks after cryoablation revealed a cryoablated mass with adjacent stranding and adherent descending colon as well as bubbles of gas in the area of stranding, the left collecting system, and the bladder. These features were consistent with a colorenal fistula at the site of previous ablation. Successful resolution of the fistula, both clinical and radiological, was achieved following a complete conservative non-interventional out-patient approach. No ureteric stent or surgical intervention was employed.

**Conclusions:**

In the absence of severe symptoms or sepsis, complete conservative management of a colorenal fistula complicating percutaneous cryoablation of renal tumors should be considered prior to interventional stenting or resectional surgery.

## Introduction

Colorenal fistula complicating percutaneous cryoablation of renal cell carcinoma has been reported in the literature on two previous occasions [1,2]. However, the most appropriate method of treatment is unclear. We present a patient with a colorenal fistula complicating cryoablation of renal tumors who had his fistula successfully treated with a complete conservative approach. No ureteric stent or surgical intervention was employed.

## Case presentation

A 62-year-old Caucasian man presented with pneumaturia and left flank pain six weeks after ultrasound-guided percutaneous cryoablation of two lesions in the anterior aspect of the mid pole and posterior upper pole of the left kidney 14 years after previous partial left nephrectomy for a left renal cell carcinoma. Our patient had an extensive medical history related to Von Hippel-Lindau disease, including previous right adrenalectomy for a phaeochromocytoma, right partial nephrectomy for renal cell carcinoma, posterior fossa surgery for a cerebellar hemangioblastoma, left partial nephrectomy for a renal cell carcinoma, and multiple pulmonary emboli resulting in the insertion of an inferior vena cava filter.

**Figure 1:**
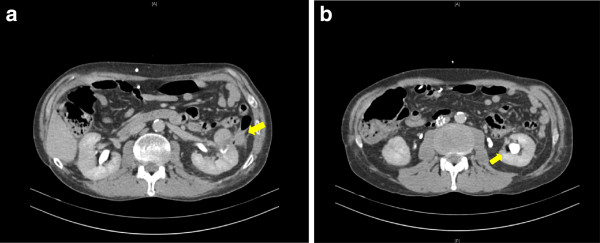
**(A, B) Computed tomography scan of the colorenal fistula at presentation shows bubbles of gas in the area of stranding and the left collecting system** .

On presentation, our patient was afebrile, and urine analysis revealed a sterile pyuria. A biphasic contrast-enhanced computed tomography (CT) scan revealed a cryoablated mass with adjacent stranding of the descending colon adherent to the mass as well as bubbles of gas in the area of stranding, the left collecting system, and the bladder (Figure 1). A diagnosis of colorenal fistula was made.

**Figure 2:**
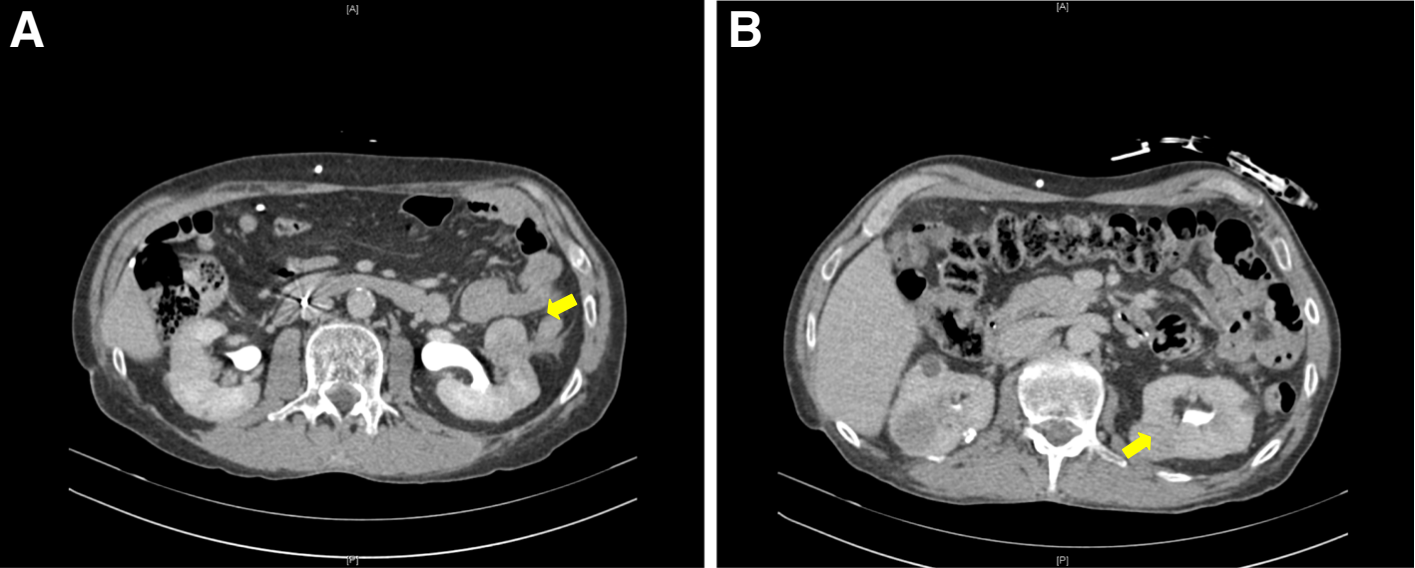
**(A, B) Computed tomography scan shows complete resolution of the fistula after treatment, resolution of pericolic fat stranding, and disappearance of gas bubbles** .

Having considered possible ureteric stenting [1] or resectional surgery [2], we decided to treat our patient with a completely conservative non-interventional out-patient approach. He was given a two-week course of antibiotics: 625mg of co-amoxiclav three times a day. All symptoms of pneumaturia and pain resolved within one month, and a follow-up CT scan confirmed complete resolution of the colorenal fistula (Figure 2). Our patient was followed up for 18 months after resolution of the fistula.

## Discussion

The incidence of renal cell carcinoma is increasing, and there has been a shift toward utilization of nephron-sparing surgery when feasible. Renal cryoablation is associated with higher local retreatment rates in comparison with radical or partial nephrectomy, although intermediate-term oncological outcomes suggest that disease-specific survival approaches that of resectional surgery [3].

Despite this change in treatment approach, colorenal fistula complicating renal cryoablation has been reported on only two occasions [1,2]. During the procedure of cryoablation, the colon is typically hydrodissected to reduce the risk of colonic injury. Therefore, it is possible to inadvertently puncture the colon with an applicator or extension of the ablation zone. Perforation may be followed by fistula or abscess formation or both [4].

Diagnosis of colorenal fistula should be based on clinical and radiological evidence. In this case, a CT scan was diagnostic [5]. There are no available data to suggest the optimal treatment option. Wysocki *et al.*[2] reported successful surgical treatment involving nephrectomy, colectomy, and end colostomy. Vanderbrink *et al.*[1] reported successful symptom resolution after placement of an internal ureteric stent. Our experience suggests that, in the absence of symptoms indicating that surgery may be required, such as intestinal obstruction, bleeding, sepsis, or renal failure [6], symptoms of a colorenal fistula may resolve spontaneously with complete conservative non-interventional out-patient management. No ureteric stent or surgical intervention was necessary.

In this case, careful follow-up and close communication with our patient allowed successful conservative treatment. Our patient had no recurrence of his colorenal fistula over a follow-up period of 18 months. This avoided the need for interventional or extensive resectional procedures, which would have been potentially high risk in a patient with significant co-morbidities.

## Conclusions

Colorenal fistula complicating percutaneous cryoablation of renal tumors can be successfully treated with non-interventional conservative management.

### Consent

Written informed consent was obtained from the patient for publication of this case report and the accompanying images. A copy of the written consent is available for review by the Editor-in-Chief of this journal.

## Abbreviation

CT: computed tomography.

## Competing interests

The authors declare that they have no competing interests.

## Authors' contributions

AM wrote the original draft of the manuscript. AD completed all of the urological treatment and follow-up of the patient and revised the manuscript. JD treated the patient clinically, supervised the project, and revised the manuscript. All authors read and approved the final manuscript.
